# ANXA2 Silencing Inhibits Proliferation, Invasion, and Migration in Gastric Cancer Cells

**DOI:** 10.1155/2019/4035460

**Published:** 2019-05-02

**Authors:** Rui Xie, Jia Liu, Xuefeng Yu, Chunfeng Li, Yufeng Wang, Wei Yang, Jiahe Hu, Ping Liu, Hong Sui, Peiqiang Liang, Xinyan Huang, Lijuan Wang, Yuxian Bai, Yingwei Xue, Jiuxin Zhu, Tianyi Fang

**Affiliations:** ^1^Harbin Medical University Cancer Hospital, 150 Haping Road, Harbin 150081, China; ^2^Department of Pharmacology (State-Province Key Laboratories of Biomedicine-Pharmaceutics of China, Key Laboratory of Cardiovascular Medicine Research, Ministry of Education), College of Pharmacy, Harbin Medical University, Harbin 150081, China

## Abstract

Annexin A2 (ANXA2) has been well known to associate with the progress of malignant tumor. However, the biological behavior of ANXA2 in gastric cancer (GC) remains unclear. We made a hypothesis in transcriptome level from TCGA datasets. Then, we used immunohistochemical staining to quantify the expression level of ANXA2 protein in GC tissues compared with adjacent tissues. Quantitative real-time PCR and western blot were used for analyzing ANXA2 expression in human GC (SGC-7901, MKN-45, BGC-823, and AGS) cell lines. We investigated the effect of a lentivirus-mediated knock-down of ANXA2 on the proliferation, invasion and migration of gastric cancer AGS cells. Cell proliferation was examined by MTT and colony formation tests. Cell apoptosis and cycle were measured by flow cytometry. Migration and invasion were detected by transwell assay. We found that high expression of ANXA2 can increase the mobility of cancer cells from TCGA datasets. ANXA2 was upregulated in GC tissues compared with adjacent tissues. AGS cell line displayed significantly higher expression of ANXA2 among the four GC cell lines. In addition, ANXA2 silencing led to a weakened ability of proliferation, invasion, and migration in GC cells; targeting of ANXA2 may be a potential therapeutic strategy for GC patients.

## 1. Introduction

Gastric cancer (GC) is the fifth highest incidence malignant tumor in the world and the third dominant cause of cancer death, with a 5-year survival rate of only 20% to 25% worldwide [1, 2]. Despite the improvement of therapeutic methods with surgical resection, chemotherapy, radiotherapy, immunotherapeutic strategies, and targeted therapies, invasion and metastasis lead to the poor prognosis of GC patients and have become a significant clinical challenge [3–7]. Therefore, finding new molecular markers which are related to metastasis and poor outcome may contribute to affording new insights into diagnostic decision and novel therapies for GC patients.

ANXA2 is a 36 kDa calcium-dependent phospholipid-binding cytoskeletal protein; it is also named as Annexin II, Annexin a2, p36, and lipocortin II [8]. Upregulation of ANXA2 was observed in many different cancer types, including hepatoma [9], pancreatic cancer [10], breast cancer [11], glioma [12], colorectal cancer [13], and GC [14]. ANXA2 mainly participates in cell membranes formation and takes effect on regulate cytoskeleton. The cytoskeleton changes are common in malignant transformation, adhesion, movement, and metastasis which may promote tumor cell to move [15]. Thus, targeting cancerous cells motility may be important to the treatments. However, it is difficult to investigate the exact function of ANXA2 in GC directly. With the development of transcriptomics, analysis of transcriptome sequencing data from real pathological specimens may provide a comprehensive background for us to understand the relationship between ANAX2 and GC cells.

To clarify this phenomenon, we analyzed the ANAX2 expression in GC tissues from The Cancer Genome Atlas (TCGA) database by R2 analysis platform. Gene Correlation analysis, Gene Ontology (GO) analysis, and Kyoto Encyclopedia of Genes and Genomes (KEGG) analysis were performed at the level of transcriptomics. The expression level of ANXA2 protein in GC tissues compared with adjacent tissues was evaluated. Then, we focused on the motility changes of GC cells when inhibiting ANXA2, including the function of proliferation, invasion, and migration.

## 2. Materials and Methods

### 2.1. Bioinformatics Analysis

The RNA sequencing (RNA-seq) data was downloaded from TCGA database (https://portal.gdc.cancer.gov/); it contained ANXA2 RNA expression data for human GC profiles, including 415 tissue samples. Gene Correlation analysis was performed with the R2: Genomics Analysis and Visualization Platform (http://r2.amc.nl); the genes and pathways associated with ANXA2 were tested by GO and KEGG pathway analysis. Correlation statistics were calculated using the R2 platform and* P*<0.05 was statistically significant.

### 2.2. Tissue Microarray

A GC tissue microarray (HStm-Ade180Sur-06) was obtained from Shanghai Outdo Biotech. GC was staged according to the WHO classification criteria: 90 tumor tissues with stages I to IV and paired noncancerous tissues. The present study was approved by the Institutional Review Boards of participating institutions.

### 2.3. Immunohistochemistry

Immunohistochemical staining for ANXA2 was performed by using the EnVision Detection Systems (Dako, Glostrup, Denmark) according to the manufacturer's instructions, with ANXA2 rabbit monoclonal antibody (Cell Signaling Technology, Beverly, MA, USA) at 1:2000 dilution as the primary antibody. Staining intensity of ANXA2 was graded as follows: negative staining (-), <5% of total cells; weak staining (+), 5%-15%; moderate staining (++), 16%-50%; and diffuse positive staining (+++), >50%.

### 2.4. Cell Culture

GC cell lines including SGC-7901, MKN-45, BGC-823, and AGS cells with different grade of differentiation were cultured in RPMI1640 medium supplemented with 10% fetal bovine serum, 100 U/ml penicillin, and 0.1 mg/ml streptomycin, in humidified air containing 5% CO2 at 37°C. The culture medium was replaced every other day.

### 2.5. RNA Isolation and Reverse Transcription

Total RNA was extracted from cells using TRIzol Reagent (Invitrogen, Carlsbad, CA, USA) according to manufacturer's protocol. RNA concentration and quality were determined by the NanoDrop 2000c Spectrophotometer (Thermo Scientific, MA, USA). Subsequently, cDNA was synthesized using M-MLV reverse transcriptase, dNTPs, RNase inhibitor (Promega, Madison, WI, USA), and an oligo dT purchased from Sangon Biotech (Shanghai, China).

### 2.6. Quantitative PCR (q-PCR)

Then, q-PCR was carried out using SYBR Master Mixture (TaKaRa, Ohtsu, Japan) and TaKaRa Thermal Cycler Dice Real-Time PCR System TP800. The primer sequences were as follows: 5′-GTGGTGGAGATGACTGAAGCC-3′ (sense) and 5′-CCACGGGGACTGTTATTCG-3′ (antisense) were for ANXA2 (110 bp). Glyceraldehyde 3-phosphate dehydrogenase (GAPDH) was used as an internal control; 5′-TGACTTCAACAGCGACACCCA-3′ (sense) and 5′-CACCCTGTTGCTGTAGCCAAA-3′ (antisense) were for GAPDH (121 bp). The thermal cycling conditions consisted of 1 cycle at 95°C for 30 s, followed by 45 cycles at 95°C for 5 s and 60°C for 30 s. Data were analyzed by the comparative threshold (2-∆∆Ct) method.

### 2.7. Western Blot

Proteins in cell lysates were separated using sodium dodecyl sulfate-polyacrylamide gel electrophoresis and electrophoretically transferred to polyvinylidene difluoride membranes. Proteins were probed overnight at 4°C with ANXA2 rabbit monoclonal antibody and GAPDH mouse monoclonal antibody (internal standard, Boster Ltd., Wuhan, China), followed by incubation with a horseradish peroxidase-conjugated secondary antibody (Kangcheng Bio-tech, China). Antibody binding was detected using peroxidase-conjugated secondary antibodies (Boster Ltd., Wuhan, China) for another 2 h at room temperature. The immunoreactive bands were visualized by western blot detection system ECL (Pierce Biotechnology, Rockford, IL, USA) and the intensity of the detected bands was analyzed using an Image J program.

### 2.8. Construction of Lentivirus and Infection

To knock down ANXA2 in AGS cells, a small hairpin RNA (shRNA) sequence, 5′-GGATGCTTTGAACATTGAA-3′, was designed to target the ANXA2 gene and subcloned into the GV115 lentiviral vector (GeneChem Co., Ltd., Shanghai, China). The scrambled siRNA sequence (5′-TTCTCCGAACGTGTCACGT-3′) was used as the negative control. Subsequently, the lentiviral transfer vector plasmid, packaging plasmid, and envelope plasmid were transfected into human embryonic kidney (HEK) 293T cells (American Type Culture Collection, Manassas, VA, USA) using Lipofectamine 2000 reagent (Invitrogen Life Technologies, Carlsbad, CA, USA) for 48 h. Then, cell supernatants containing lentivirus was harvested and concentrated. The virus titer was calibrated in the HEK293T cells. The lentivirus with a final concentration of 4×10^8^ TU/ml was stored at -80°C. For lentivirus infection, according to the multiplicity of infection (MOI), cells grown to 30% confluence were transfected with lentivirus containing green fluorescent protein (GFP) reporter. The cell medium was changed to complete medium after 12 h of transfection. After cultured for another 3 d, the infection rates were determined using fluorescence microscope.

### 2.9. Cell Proliferation Assay

Cell proliferation was tested by evaluating cell viability using the MTT assay. Briefly, cells seeded on 96-well plates were stained with 20 *μ*l of 5 mg/ml MTT dye (Beijing Dingguo Changsheng Biotechnology Co., Ltd., Beijing, China) per well for 4 h at 37°C. After removal of the culture medium, 150 *μ*l of dimethyl sulphoxide (DMSO, Sinopharm Chemical Reagent Co., Ltd., Beijing, China) was added to solubilize the formazan. The absorbance values were determined at a wavelength of 495 nm in a BioTek ELx800 microplate reader. Three replicate wells were measured per assay, each experiment was performed at least 3 times.

### 2.10. Colony Formation Assay

Cells were plated on 6-well plates at an initial density of 800 cells per plate. The cells were cultured for up to 14 d or allowed to grow until most of colonies reached>50 cells per colony. After washing with phosphate buffered saline (PBS), the colonies were fixed with 10% paraformaldehyde for 30-60 min, then stained with Giemsa and washed. Colony number was determined under an Olympus fluorescence microscopy equipped with MicroPublisher 3.3 RTV CCD camera.

### 2.11. Cell Apoptosis Assay

Flow cytometry was performed to detect cell apoptosis rates using Annexin V Apoptosis Detection Kit APC (eBioscience, San Diego, CA, USA) according to the manufacture's protocol. In brief, after washing the collected cells with PBS, the cells were incubated with 1×Annexin V binding buffer. Then 100 *μ*l cells (1×10^5^-1×10^6^ cells) were stained with Annexin V-APC for 10 min at room temperature in the dark, we assessed the Annexin V-positive cells by a BD FACSCalibur flow cytometer.

### 2.12. Cell Cycle Analysis

Cells in culture were collected, resuspended in ice-cold PBS and fixed with 70% of ice-cold ethanol at 4°C for at least 1 h. Then, the fixed wells were washed with PBS and stained with propidium iodide (PI, Sigma-Aldrich, St. Louis, MO, USA) and RNase A (Fermentas, St. Leon-Rot, Germany) in the dark at room temperature before analysiing the cell percentage in each cell cycle phase using a FACSCalibur instrument.

### 2.13. Matrigel Invasion Assay and Transwell Migration Assay

Cell invasion assay was tested using Biocoat Matrigel Invasion Chamber (Becton-Dickinson, Bedford, MA), a 24-well transwell unit with 8 *μ*m pore size polyethylene terephthalate membrane, according to the manufacturer's protocol. Briefly, 1×10^5^ cells in the top chamber incubated in 100 *μ*l of fetal bovine serum- (FBS-) free medium were allowed to attach to 24-well transwell plates. The lower invasion chamber contained 600 *μ*l of RPMI-1640 medium with 30% FBS. After 24 h of incubation, the cells that invaded the lower surface of the membrane were fixed and stained with Giemsa for 30 min. Under a phase-contrast microscope, we got the images of the cells in five predetermined fields at a magnification of 100 and 400. The stained cells were dissolved in 10% acetic acid [16, 17]; the invasion was calculated by measuring the optical density (OD) values at a wavelength of 570 nm (OD 570).

Cells (1×10^5^) incubated in 100 *μ*l of FBS-free medium were added to the 12-well format top chambers with 8.0 *μ*m pore size porous transparent polyethylene terephthalate membrane. The lower chamber of the 12-well format transwell contained 600 *μ*l of RPMI-1640 medium with 30% FBS. After incubation for 24 h, the cells that invaded the lower surface of the membrane were stained with Giemsa. Pictures were taken under the microscope. The stained cells were dissolved in 10% acetic acid and OD 570 was measured. The percentage of migration was evaluated by determining the OD570/MTT-OD490 ratio of the cells.

### 2.14. Wound Scratch Test

Cells in 96-well culture plates were grown in complete medium until cells reached 90-100% confluence. A wound was scratched across the diameter of each plate. The cells were washed gently using PBS to remove cell debris. Cell migration was observed by microscopy at 0, 8, and 24 h.

### 2.15. Statistical Analysis

Statistical comparisons between groups were determined by Student's t-test and chi-square test using SPSS 20.0 (SPSS Inc., Chicago, IL, USA). Statistical significance was assumed for* P*<0.05.

## 3. Results

### 3.1. Expression of ANXA2 in TCGA Database

**Figure 1:**
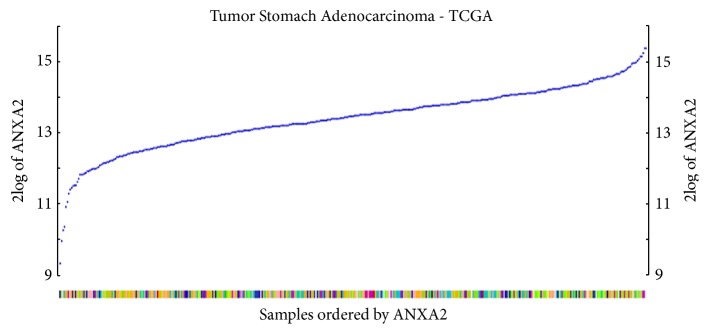
By analyzing the expression of ANXA2, we found ANXA2 was widely expressed in gastric cancer tissues. The blue points showed the expression level ANXA2. Different colors of X-Line indicated gastric cancer patients with different stages.

**Figure 2:**
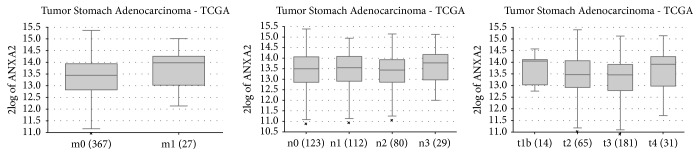
Differential expression of ANXA2 could differentiate M staging of gastric cancer patients (*P*=0.04), not T staging or N staging.

By analyzing the expression level of ANXA2 in TCGA database, we found that ANXA2 was highly homogeneously expressed in GC tissues (Figure 1). The samples were divided into T stage (T1b, 14 samples; T2, 65 samples; T3, 181 samples, T4, 31 samples); N stage (N0, 123 samples; N1, 112 samples; N2, 80 samples; N3, 29 samples); and M stage (M0, 367 samples; M1, 27 samples). Then we analyzed the expression of ANXA2 in different stages. We found that the expression level of ANXA2 in patients with distant metastasis (M1) was higher than that in nondistant metastasis patients (M0). But there was no statistical significance in T stage and N stage (Figure 2).

### 3.2. Function of ANXA2 and Its Related Genes Analyzed by KEGG and GO Analysis

Using the R2 platform analysis, 415 samples were analyzed statistically; 3323 positive genes and 5700 negative genes were significantly correlated with ANXA2 expression. The top 10 positively and negatively correlated genes were shown in Tables 1 and 2. To determine the function of ANXA2 and its related genes, we used GO analysis and KEGG analysis (Tables 3 and 4). Biological processes included structure morphogenesis and movement of cells. Among the 9023 genes associated with ANXA2, KEGG analysis found that 3006 genes were involved in 47 signaling pathways; most of them were related to pathways in cancer and regulation of actin cytoskeleton (*P*<0.005).

**Figure 3:**
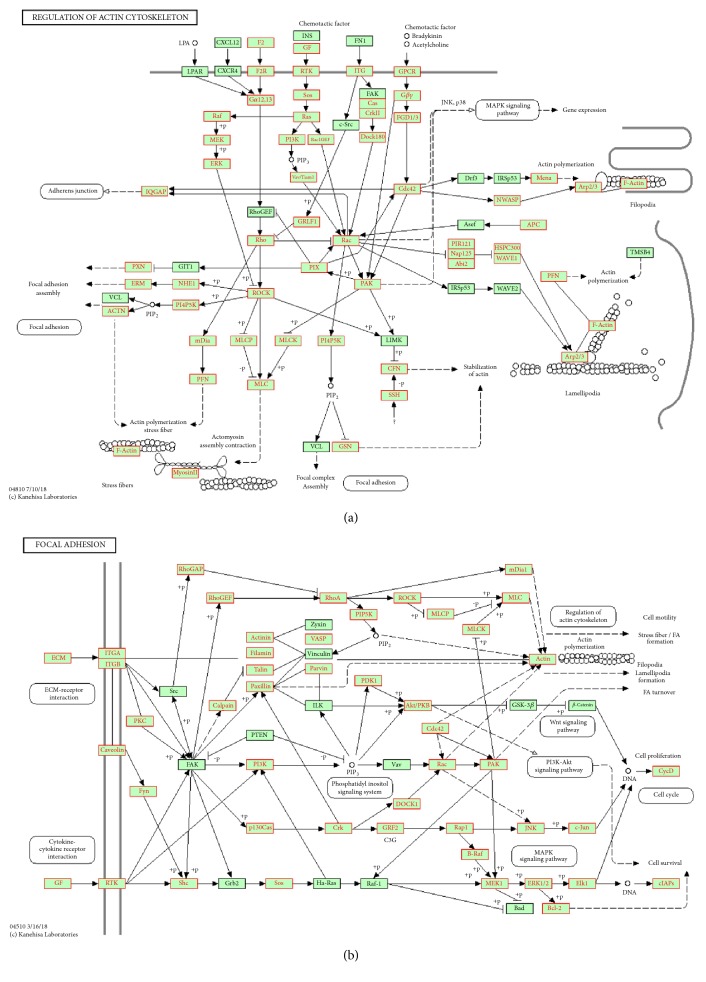
(a) By KEGG pathway enrichment analysis, ANXA2 upregulated filopodia pathway, resulting in cell metastasis. Microfilament is one component of cytoskeleton; it is mainly composed of actin. ANXA2 upregulated regulation of actin cytoskeleton pathway and led to the rise of cell deformability. (b) By KEGG pathway enrichment analysis, ANXA2 upregulated focal adhesion pathway, resulting in cell motility, proliferation and survival.

Among the genes coexpressed with ANXA2, we found that the signaling pathways ‘Focal adhesion' and ‘Regulation of actin cytoskeleton' were activated, which was the same as we anticipated (Figure 3). This actin polymerization may change cytoskeleton. On the other hand, the filamentous filopodia may help the movement of tumor cells.

### 3.3. Expression of ANXA2 in GC Tissues and Adjacent Tissues

**Figure 4:**
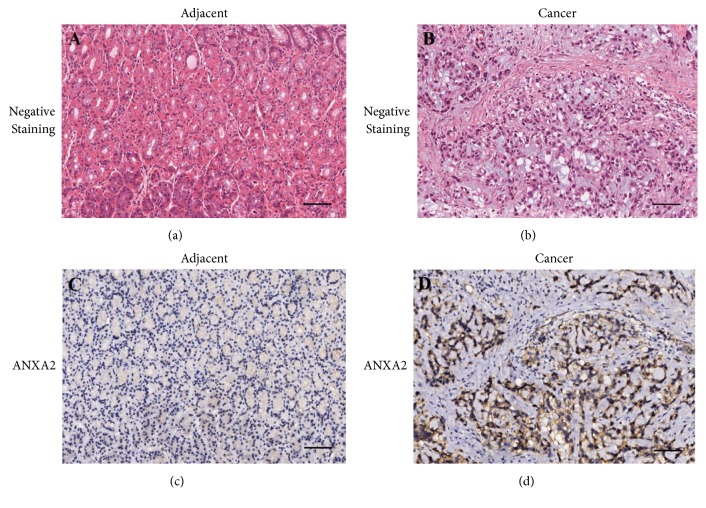
Immunohistochemical staining of ANXA2 was upregulated in gastric cancer tissue than adjacent tissue. Negative staining of (a) adjacent tissue and (b) gastric cancer tissue. ANXA2 expression of (c) adjacent tissue and (d) gastric cancer tissue (magnification, 100×, scale bar, 50 *μ*m).

Our experiments summarized the different expression level of ANXA2 in GC tissues and adjacent tissues based on immunohistochemical analysis. ANXA2 mainly expressed in GC tissues with obvious brownish yellow particles (Figure 4(d)). Negative ANXA2 expression was detected in 29 (32.2%) of the 90 GC tissues samples, 6 samples (6.7%) were hadropositive and 25 samples (27.8%) were positive ANXA2 expression (Table 5(a)). The remaining samples were weakly positive. In contrast to GC tissues, 91.1% of the adjacent tissues showed negative ANXA2 expression (Figure 4(c)). The data showed that the distribution of ANXA2 expression in GC and adjacent tissues was statistically significant (*P*<0.001, Table 5(b)).

### 3.4. Expression of ANXA2 in Different GC Cell Lines

**Figure 5:**
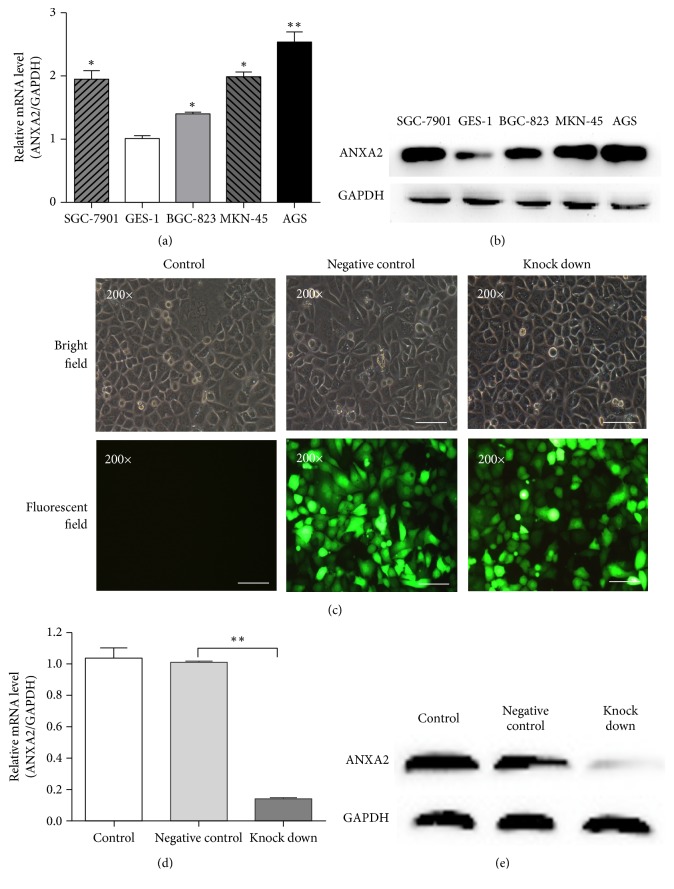
ANXA2 siRNA suppressed the ANXA2 mRNA and protein expression levels in gastric cancer cell lines. (a) Quantitative real-time PCR analyzed ANXA2 mRNA levels in different gastric cancer cell lines including SGC-7901, GES-1, BGC-823, MKN-45, and AGS. ∗*P<*0.05, ∗∗*P<*0.01 compared to GES-1. (b) The expression level of ANXA2 protein in SGC-7901, GES-1, BGC-823, MKN-45, and AGS cells was determined using western blot. GAPDH was utilized as the internal control. (c) Bright and GFP fluorescent field of AGS cell 72 h after infection with negative control (middle panel) or lentivirus containing ANXA2-RNAi (knock-down, right panel), magnification, 200×, scale bar, 100 *μ*m. (d) Quantitative real-time PCR assessment of ANXA2 mRNA levels in AGS siRNA-infected cells compared to control and negative control cells, ∗∗*P*<0.01. (e) Western blot analysis of ANXA2 protein expression in ANXA2 knock-down cells compared to control and negative control cells.

Expression level of ANXA2 mRNA in human GC cell lines with different grade of differentiation was examined by quantitative real-time PCR. The results showed that ANXA2 mRNA was positively expressed in four human GC cells (SGC-7901, BGC-823, MKN-45, and AGS cells), the highest expression of ANXA2 mRNA was tested in AGS cells (Figure 5(a)). Consistent with mRNA expression level, AGS cell had the highest expression level of ANXA2 protein detected by western blot (Figure 5(b)).

### 3.5. Expression of ANXA2 mRNA and Protein in AGS Cells

To suppress ANXA2 expression, AGS cells were infected with a lentivirus which expressed ANXA2-spcific siRNA and GFP. After 72 h infection, more than 80% of cells expressed GFP, indicating successful infection (Figure 5(c)). ANXA2 mRNA (Figure 5(d)) and ANXA2 protein expression (Figure 5(e)) in AGS cells infected with lentivirus expressing ANXA2 siRNA (knock-down group) were significantly lower than in cells infected with lentivirus expressing scrambled siRNA (negative control group) and AGS cells (control group). These results demonstrated that siRNA directed towards ANXA2 was efficient in specifically knocking down the ANXA2 gene in AGS cells.

### 3.6. The Function of ANXA2 on Proliferation in AGS Cells

**Figure 6:**
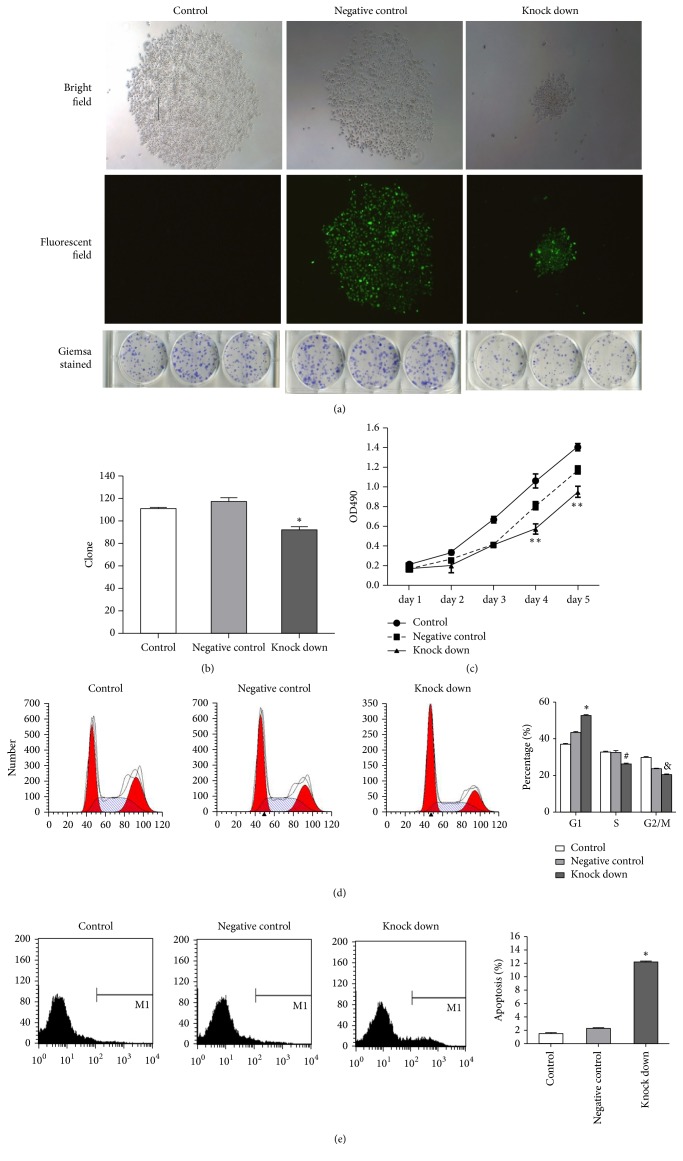
ANXA2 silencing inhibited AGS cell growth. (a) Representative size of cell colonies, bright field (upper panel), fluorescent field (middle panel), and Giemsa stained (lower panel),* P*<0.05. (b) The number of cell colonies in control, negative control, and ANXA2 knock-down cells, ∗*P*<0.05. (c) Using MTT assay, the relative AGS cell proliferation pattern at different time points was investigated, ∗*P*<0.05, n=5. (d) The ratio of cells at different cell cycle phases in control, negative control, and ANXA2 knock-down groups. (e) The cell apoptotic rate in control, negative control, and ANXA2 knock-down groups using flow cytometry, ∗*P*<0.05, n=3.

As illustrated in Figures 6(a) and 6(b), compared with control and negative control groups, numbers of cells colony formation capacity significantly reduced in ANXA2 knock-down group. The MTT assay showed obviously less proliferation in ANXA2 knock-down group at 4 and 5 d after infection than other groups (Figure 6(c)). These findings totally revealed that ANXA2 was essential for AGS cell proliferation. ANXA2 silencing caused G1 phase arrest of AGS cells and a decline in the number of cells in the S and G2/m phases compared to negative control and control cells (Figure 6(d)). The rates of apoptosis in ANXA2-silencing cells were appreciably increased (Figure 6(e)).

### 3.7. The Function of ANXA2 on Invasion and Migration in AGS Cells

**Figure 7:**
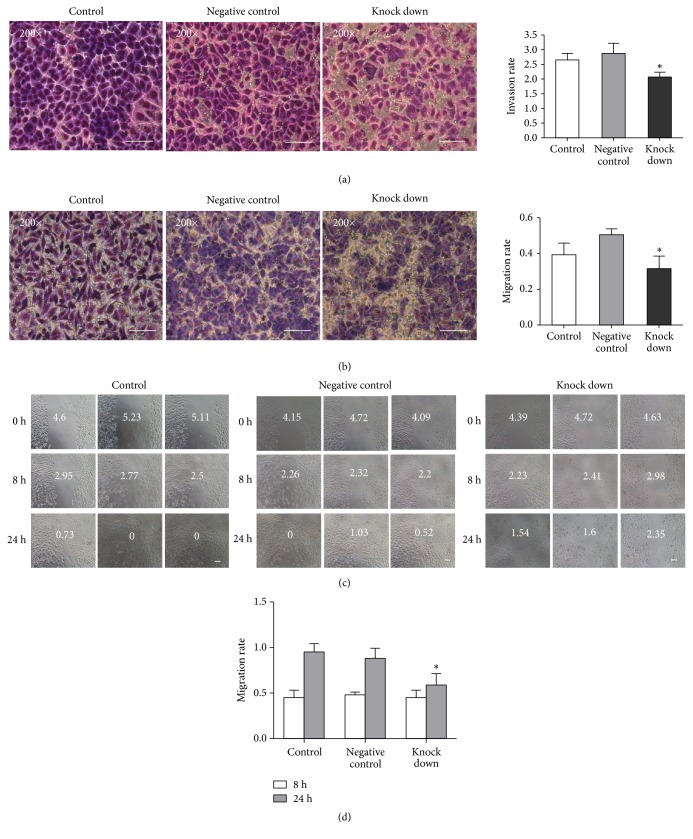
ANXA2 silencing inhibited invasion and migration rate of AGS cells. (a) Giemsa staining and compared with negative control group, the invasion rate in knock-down group was decreased, ∗*P*<0.05, n=3, scale bar, 100 *μ*m. (b) Giemsa staining and compared with negative control group, the migration rate in knock-down group was decreased, ∗*P*<0.05, n=3, scale bar, 100 *μ*m. (c) Picture of cell scratch, scale bar, 100 *μ*m. (d) In 8 h, the migration rate has no significant difference in control, negative control, and knock-down groups. Compared with control group and negative control group, at 24 h, the migration rate of knock-down group was lower, ∗*P*<0.05, n=3.

Matrigel invasion chamber, transwell, and scratch assay provided evidence that ANXA2 silencing inhibited invasion and migration ability in ANXA2 knock-down group than in control and negative control groups (Figures 7(a) and 7(b)). The results showed that the migration rate of ANXA2 knock-down group cells was significantly decreased at 24 h than in negative control and control cells (Figures 7(c) and 7(d)).

## 4. Discussion

GC is a heterogeneous disease with a high mortality rate. Its poor prognosis is mainly contributed by extensive invasion and metastasis [18]. The mechanism of tumor metastasis is complex. It includes cancerous cells away from their primary tumor, the intravasation into the bloodstream or lymph nodes, the transit through the tumor microenvironment, and the aggregation to secondary tissues. Cancer cell migration plays an important role in the metastatic process. However, the specific reasons have not been clarified yet. Therefore, in-depth research on genes related to invasion and metastasis of GC is crucial.

ANXA2 is overexpressed in most tumor tissues and acts as a pivotal part in tumor development. Prior research generally confirms that the expression level of ANXA2 has a close relation with progression of tumors [19–21]. Zhang et al. [14] prove that expression level of ANXA2 mRNA is higher in GC tissues than nontumor tissues. Besides, as the expression level of ANXA2 increases, the degree of pathological differentiation of GC cells decreases [22]. Consistent with previous study, our research demonstrated that ANXA2 was significantly enhanced in GC tissues compared with adjacent tissues.

Previous reports have shown the effect of ANXA2 in many cancerous cells. For example, ANXA2 may promote the progression and invasion of human lung cancer cell [23]. With the upregulation of ANXA2, the potential ability of metastatic and invasive is increased in hepatoma cell, shRNA-mediated ANXA2 silencing significantly inhibits cell invasion, migration, and tumorigenic potential [24]. Similar studies have studied ovarian cancer cell [25], glioma cell [12], and breast cancer cell [26]. However, there is no relevant study on biological behavior of ANXA2 in GC cells.

We first analyzed the expression of ANXA2 in GC tissue from TCGA database; it made a new view for the research of GC patients. Theoretically, the large size of the RNA-Seq dataset may improve the reliability of the results. Thus, data analysis depending on RNA-Seq can provide a more realistic context for us. It was interesting that we first verified our conjecture from TCGA database. The high expression of ANXA2 activated tumor cells to generate cytoskeleton, acted a positive role in proliferation of GC cells. On the other hand, the high expression of ANXA2 activated the pathway for tumor cells to grow filopodia, it provided energy for cancerous cells to move themselves. From this perspective, we explained why the high expression of ANXA2 indicated poor prognosis of GC.

In our study, AGS cell line displayed the highest expression level of ANXA2 among the four kinds of human GC cell lines and it was selected as a cell model for subsequent experiments. To elucidate roles of ANXA2 in GC cell growth, AGS cells were infected with a lentivirus expression ANXA2-spcific siRNA and GFP, it laid the foundation for the further experiments.

Our results observed that the proliferation and colony formation ability of lentivirus-mediated Annexin A2 knock-down cells were suppressed appreciably; it prompted us to consider that ANXA2 might act as a positive role in proliferation of GC cells in vitro. ANXA2 knock-down cells showed a significant more proportion of cells in the G1 phase and a decline number in the S and G2/m phases compared to negative control or control cells. Similarly, in previous study, non-small cell lung cancer cell lines with ANXA2 silencing showed inhibition both in tumor growth and cell proliferation and also induced cell cycle arrest at the G2 phase [27]. Apoptosis rates apparently increased in ANXA2 knock-down cells. Abnormal acceleration of cell cycle and inhibition of apoptosis are important causes of the tumor cell growth. These results suggested that ANXA2 was a promising target for inhibiting GC cell proliferation possible by blocking the cell cycle and promoting cell apoptosis.

Invasion and metastasis as the important biological behaviors of malignant tumor are the leading causes for poor prognosis of GC patients [28]. Numerous factors contributed to regulate invasion and metastasis and other biological behaviors in tumor cells. Our experiment showed that knocking down ANXA2 expression inhibited invasion and migration ability of AGS cells to achieve the purpose of delaying the progression of GC.

In our previous published study, we used gene expression microarray and detected 100 differentially expressed genes including 58 upregulated and 42 downregulated human genes. According to all pathway genetic information in KEGG and BIOCARTA, we did the enrichment analysis of the difference of gene; the important ANXA2 related genes including FGA, FGB, SERPINB2, CD55, PLAUR, MET, RAP1A, and ETS1 were screened. Western blot showed that after silencing of ANXA2, RAP1A, and MAP2K1 protein expression decreased in AGS cells. We speculated that RAP1A and MAP2K1 might be potential downstream regulating genes of ANXA2 taking part in MAPK signaling pathway in AGS cells [29].

Identifying target points of genes related to proliferation, invasion, and migration of GC is essential to consummate traditional treatments and improve prognosis of patient. Our experimental results provided further insights into the research progression on biological behavior of GC. It could lead researchers to taking serious consideration of the potential benefit of ANXA2 silence as an effective antitumor therapeutic tool.

## 5. Conclusions

In conclusion, our results presented a compelling evidence for the role of ANXA2 in biological behavior of GC. Analyzing the TCGA datasets, we found that ANXA2 promoted cell migration and proliferation in transcriptome level. The ANXA2 protein and mRNA expression in GC tissues were significantly upregulated. Lentivirus-mediated ANXA2 silencing affected the biological behavior of AGS cells through inhibiting the proliferation, invasion, and migration, promoting apoptosis, and arresting cell cycle.

## Figures and Tables

**Table 1:** Positive correlated genes with ANXA2.

Gene	*R*-value	*P*-value

ANXA2P2	0.910	<0.005
S100A6	0.683	<0.005
S100A16	0.682	<0.005
S100A11	0.637	<0.005
GPRC5A	0.627	<0.005
S100A10	0.615	<0.005
SFN	0.602	<0.005
EZR	0.598	<0.005
PLEK2	0.593	<0.005
EPHA2	0.583	<0.005

**Table 2:** Negative correlated genes with ANXA2.

Gene	*R*-value	*P*-value

CBFA2T2	-0.561	<0.005
CHD6	-0.559	<0.005
ZFHX3	-0.540	<0.005
BSN	-0.529	<0.005
NTN3	-0.513	<0.005
ZNF445	-0.508	<0.005
IQSEC1	-0.505	<0.005
CBX6	-0.486	<0.005
SEZ6	-0.481	<0.005
IRF2BPL	-0.480	<0.005

**Table 3:** GO analysis of ANXA2 in the database.

Biological process	No. of genes	*P*-value	Go path no.

membrane-bounded organelle	5877	<0.005	43227
anatomical structure morphogenesis	1332	<0.005	9653
cell morphogenesis	553	<0.005	902
protein binding	5369	<0.005	5515
movement of cell or subcellular component	1006	<0.005	6928
cytoskeleton organization	673	<0.005	7010

**Table 4:** KEGG analysis of ANXA2 in the database.

Biological process	No. of genes	*P*-value	Related genes
Pathways in cancer	224	<0.005	APPL1, FZD10, LPAR6, GNA13, CXCL12, ABL1, PIK3R5, AKT3, MTOR, STAT5A, ARAF, BCL2, SMAD4, TGFBR1, MSH3, PIAS2…

Focal adhesion	122	<0.005	EGF, EGFR, PIK3R5, ITGA11, LAMC3, PAK4, DOCK1, ROCK1, GRF2, MAPK8, JUN, BLC2, ELK1, BRAF, RAPGEF1, CRK, SOS1…

HIF 1 signaling pathway	67	<0.005	AKT2, AKT3, ANGPT1, ARNT, BCL2, CAMK2A, CAMK2B, EGLN1, ENO3, EP300, EPO, PIK3CD, PIK3CG, TEK, TF, VHL…

Proteoglycans in cancer	116	<0.005	ELK1, ROCK1, TIAM1, ITPR1, DROSHA, HOXD10, PIK3R5, AKT3, PDPK1, MTOR, PDCD4, COL21A1, ARAF, HGF, FRS2, SOS1, WNT16…

Regulation of actin cytoskeleton	118	<0.005	F2, GNA13, ARAF, PIK3R5, SOS1, EGFR, EGF, ITGA11, CHRM1, FGD1, VAV3, MSN, ACTN4, MYLK, MYH9, CFL1, ENAH, WASF1…

Central carbon metabolism in cancer	42	<0.005	AKT2, FGFR1, FLT3, GCK, KIT, MTOR, NRAS, NTRK1, NTRK3, PDGFRA, PFKM, PGAM2, PIK3CD, RET…

Vascular smooth muscle contraction	76	<0.005	ACTA2, ADRA1A, AGTR1, BRAF, CACNA1C, EDNRA, GNA12, GNAS, ITPR1, KCNMA1, MRVI1, PLA2G12A, PLCB4, RAMP3, ROCK1…

∗For the “Biological process” column, we showed the function of ANXA2 in gastric cancer tissue. The “No. of genes” column showed the number of genes that enriched in corresponding pathways, and the details were in the “Related genes” column. Correlation statistics were calculated by the R2 platform.

**(a):** 

Tissue type	Number of cases	ANXA2 expression			
		Negative (-)	Weakly positive (+)	Positive (++)	Hadro-positive (+++)

Adjacent	90	82 (91.1%)	4 (4.4%)	3 (3.3%)	1 (1.1%)
Cancer	90	↓ 29 (32.2%)	↑ 20 (22.2%)	↑ 25 (27.8%)	↑ 6 (6.7%)

∗↓ Decreased ANXA2 expression in gastric cancer tissue compared to adjacent tissue.

↑ Enhanced ANXA2 expression in gastric cancer tissue compared to adjacent tissue.

**(b):** 

Cancer	Total Number	Adjacent		*P-*value
		Negative	Total positive	

Negative	29	28	1	<0.001
Positive	61	54	7	

## Data Availability

Figures 1, 2, and 3 data used to support the findings of this study have been deposited in the TCGA repository.

## Abbreviations

ANXA2: Annexin A2
GC: Gastric cancer
TCGA: The Cancer Genome Atlas
GO: Gene Ontology
KEGG: Kyoto Encyclopedia of Genes and Genomes
q-PCR: Quantitative PCR
PBS: Phosphate buffered saline
OD: Optical density.
